# Editorial: Advances in color science: From color perception to color metrics and its applications in illuminated environments

**DOI:** 10.3389/fpsyg.2023.1142153

**Published:** 2023-01-31

**Authors:** Yandan Lin, Wei Xu, Qiang Liu

**Affiliations:** ^1^School of Information Science and Technology, Institute for Electric Light Sources, Fudan University, Shanghai, China; ^2^Institute for Six-sector Economy, Fudan University, Shanghai, China; ^3^Shanghai Professional Technical Service Platform for Intelligent Vision & Human Factors Engineering, Shanghai, China; ^4^Research Center of Graphic Communication, Printing and Packaging, Wuhan University, Wuhan, China

**Keywords:** color vision, color science, color metrics, color preference, color perception

Color science is an inherently interdisciplinary area of research. It examines the perception of color by the human eye and brain, the origin of color in materials, color theory in art, and the physics of electromagnetic radiation in the visible range. Nowadays, on a basic research level, the most fundamental processing stages in color vision and color perception remain unexplained. At the same time, new areas in the study of applied color science have emerged in just the past decade, examining new advances in cameras, displays, sensors, virtual reality, augmented reality, mixed reality, smart lighting, and so on.

With the purpose of presenting the frontier knowledge in color science, both theoretical and applied, this Research Topic covers a total of 21 original articles, which were published from 21 Jun 2018 to 15 Dec 2022. The series is organized into three sections: color perception and psychological effects, color management in a broad sense, and spectral reconstruction.

## Color perception and psychological effects

All objects have color but bring different perceptions to the human eye or brain. This subjective perception varies with individual differences, social backgrounds, and the properties of the observed objects. Some psychological theories suggest that color, like other biologically-derived signals, should be reliably paired with an emotion, and that colors should differentiate across emotions. Such emotions include both pure color-emotion pairs (Fugate and Franco) and the effect of objects with color on individual psychological preferences.

The first area is the effect of individual differences on color perception. Common factors include age, gender, visual impairment, and visual acuity. Lian et al. conducted a study on currently controversial blue-light blocking spectacle lenses to assess their long-term effects on contrast perception in adults under different lighting conditions. The authors conclude that wearing blue-light blocking lenses had no clinically significant effect on adults' long-term contrast perception under scotopic or photopic conditions, or on glare, which contradicts the findings of some previous studies. The possible reason for the contradictory results is the difference in age of the participants.

How color affects the perception of social status in various cultural contexts was also studied (Wu et al.). The authors show that participants associate with the red color with logos of high-status stimuli more quickly than the red color and logos of low-status stimuli, using the implicit association test paradigm in seven tests. Additionally, the authors demonstrate that in different civilizations, notably China and the United Kingdom, the color red is associated with a high social rank. This finding suggests that color cognition is at least somewhat culturally ubiquitous.

Finally, there are several studies on the influence of the properties of the observed objects on color perception and psychological preference. In particular, for the object observed, some of its properties such as shape, roughness, and gloss (Honson et al.) affect the observer's subjective color perception, and its objective color in turn affects the perception of other properties of the object, such as implied social status (Wu et al.), the taste of foods (Wang and Li), the discount depth in a price promotion context (Kim and Jang), likability perception of CG character (Wang M. et al.), propensity to eat (Schlintl and Schienle), and facial expression perception (Minami et al.). At the same time, this difference in color perception can lead to different psychological preferences in specific scenarios, such as in-vehicle lighting (Weirich et al.), urban street lighting with different color temperatures (Hao et al.), and costume, food, artware, and skin lighting (Deng et al.).

In addition to studies on a single color, this section also contains studies on the modeling of color harmony based on multi-color feature extraction (Wang, Liu, Jiang, et al.) as well as the perceptual attributes of multiple colors and combined tones (Wang, Liu, Lan, et al.).

## Color management in a broad sense

Color management is the controlled conversion between the color representations of various devices. Although its main goal is to obtain a good match across color devices, in a broad sense, it also involves digitization (Su et al.), reproduction (Li et al.), correction, and optimization (Liu Z. et al.) of color.

The dyeing industry is closely related to color reproduction technology. Li et al. select the two-constant Kubelka–Munk theory to investigate the feasibility of minimizing and optimizing learning samples for the theory, and find that two samples, namely, a mas stone consisting of 100% pre-colored fiber and a tint mix of 40% pre-colored fiber and 60% white fiber, are enough to determine the absorption and scattering coefficients of a pre-colored fiber. In addition, the authors also explore optimal samples for the single-constant Kubelka-Munk theory.

Restoring the accurate or realistic color of cultural heritage objects during the archiving process is a significant challenge for imaging technology. Su et al. focus on color reproduction in silk fabrics. To overcome the disadvantage of applying traditional color charts to silk fabrics with high gloss in the conventional color management process, the authors specifically organize a unique “Qianlong Palette” color chart, consisting of 210 silk fabric samples, and compare the performance of different mapping methods on these two kinds of color charts. Liu Z. et al. present an improved color correction method for color cast images that makes the color appear more realistic. It is based on a computational model of the human visual system that perceives objects by color constancy theory and realizes illumination non-uniformity compensation and chromaticity correction for color cast images by taking the color stability of some pigments into account.

After obtaining digital images of ancient mural images, Liang, Liu, et al. explore an optimized method for segmentation based on super-pixel algorithms, improving digital virtual restoration.

## Spectral reconstruction

The surface spectral reflectance of an object is the most essential description of its surface color and is not disturbed by color metamerism. At the same time, it is also the key factor for material analysis and has been proven beneficial in many fields, including color rendering, composition analysis, and substance identification. The acquisition of spectral reflectance is the basis of its numerous applications.

Xiong et al. propose an optimized method based on dynamic partitional clustering for the recovery of spectral reflectance from camera response values. A combination of dynamic and static clustering is used to produce dynamic clustering subspaces, and the Euclidean distance weighted and polynomial expansion models in the clustering subspace are adaptively applied to improve the accuracy of spectral recovery (Xiong et al.). Liu D. et al. propose an improved sequential adaptive weighted spectral estimation method to explore the feasibility of using a mobile phone camera as a new spectral imaging device to obtain the raw responses of samples for spectral estimation. Liang, Xin, et al. propose an optimized method for spectral reconstruction based on data augmentation and attention mechanisms using the current deep learning-based spectral reconstruction framework. The proposed method is exposure invariant and adapts to the open environment in which the light is easily changed, and the illumination is non-uniform. Thus, the robustness and reconstruction accuracy of the spectral reconstruction model in practical applications are improved (Liang, Xin, et al.).

## Author contributions

All authors contributed to the article and approved the submitted version.

